# The effects of young and aged, male and female megakaryocyte conditioned media on angiogenic properties of endothelial cells

**DOI:** 10.18632/aging.206077

**Published:** 2024-11-22

**Authors:** Murad K. Nazzal, Hanisha L. Battina, Nikhil P. Tewari, Sarah L. Mostardo, Rohit U. Nagaraj, Donghui Zhou, Olatundun D. Awosanya, Saveda K. Majety, Sue Samson, Rachel J. Blosser, Ushashi C. Dadwal, Patrick L. Mulcrone, Melissa A. Kacena

**Affiliations:** 1 Department of Orthopaedic Surgery, Indiana University School of Medicine, Indianapolis, IN 46202, USA; 2 Richard L. Roudebush Veterans' Administration Medical Center, Indianapolis, IN 46202, USA

**Keywords:** bone marrow endothelial cells, megakaryocyte, conditioned media, aging, angiogenesis

## Abstract

With aging, the risk of fractures and compromised healing increases. Angiogenesis plays a significant role in bone healing and is impaired with aging. We have previously shown the impact of megakaryocytes (MKs) in regulating bone healing. Notably, MKs produce factors known to promote angiogenesis. We examined the effects of conditioned media (CM) generated from MKs derived from young (3–4-month-old) and aged (22–24-month-old), male and female C57BL/6J mice on bone marrow endothelial cell (BMEC) growth and function. Female MK CM, regardless of age, caused a >65% increase in BMEC proliferation and improved vessel formation by >115%. Likewise, young male MK CM increased vessel formation by 160%. Although aged male MK CM resulted in >150% increases in the formation of vascular nodes and meshes, 62% fewer vessels formed compared to young male MK CM treatment. Aged female MK CM improved migration by over 2500%. However, aged female and male MK CM caused less wound closure. MK CM treatments also significantly altered the expression of several genes including PDGFRβ, CXCR4, and CD36 relative to controls and between ages. Further testing of mechanisms responsible for age-associated differences may allow for novel strategies to improve MK-mediated angiogenesis and bone healing, particularly within the aging population.

## INTRODUCTION

Bone fractures are the most common musculoskeletal condition requiring hospitalization in Medicare enrollees aged 65 and older [1–4]. Furthermore, this demographic has been shown to have a decreased capacity to heal from fractures, leading to higher odds than the general population of requiring re-hospitalization due to delayed healing or nonunion of a bone fracture. Complications stemming from inadequate healing can result in more severe and systemic consequences for this population, such as incapacitation [1, 5, 6].

Normal fracture healing consists of 4 stages – hematoma formation, fibrocartilaginous callus formation, bony callus formation, and bone remodeling. Early healing is accomplished by a robust inflammatory response that recruits skeletal tissue progenitor cells that develop into a soft, cartilaginous, and avascular callus [7, 8]. Vascularization initiates the hardening of the soft callus into eventual bone through endochondral ossification by facilitating infiltration of necessary nutrients and cell types such as chondrocytes and osteoblasts [9]. The last step is characterized by remodeling of the callus through alternating osteoclastic and osteoblastic processes to re-establish normal trabecular bone structure and hematopoiesis [10]. Many age-related changes in this process have been associated with impaired and delayed fracture healing in elderly animals, such as a decrease in the proliferation and differentiation capacity of osteochondral stem cells and a delay in the deposition of cartilage matrix [11–14]. Changes that occur during the vascularization step due to aging are also an established contributor to delayed fracture healing in this population. Furthermore, these changes are potentially the most impactful considering angiogenesis is a key rate-limiting factor for bone repair [15, 16]. Indeed, fracture patients with a pre-existing vascular injury or disease can incur delayed healing or non-union at rates as high as 46%, illustrating the importance of angiogenesis in the healing process [17]. Generally, vascular perfusion of the skeleton decreases with age [18], and elderly rats display significantly decreased patency of bone marrow (BM) blood vessels compared to younger rats [19]. In fracture calluses, young mice have a higher surface density of blood vessels compared to elderly mice, which is partially attributed to an earlier induction of vascular endothelial growth factor (VEGF) and hypoxia-inducible factor (HIF)-1α production [20]. While VEGF and HIF-1α are amongst the most well-known angiogenic molecules, other factors such as fibroblast growth factor (FGF), transforming growth factor (TGF)-β, sirtuin 1 (SIRT1), and bone morphogenetic protein (BMP)-2 have also been characterized as angiogenic molecules that play a role in fracture healing and bone regulation [21–26]. Currently, only a few growth factors such as BMP-2, BMP-7, and platelet-derived growth factor (PDGF) are FDA-approved for assisting in bone formation and bone graft substitutions, with each having their own unique advantages and disadvantages [27, 28]. For example, BMP-2 administration at the fracture callus site has been shown to improve both fracture healing time and callus vascularization in mice but increases the risk of notable side effects such as systemic inflammation, ectopic bone formation, wound complications, and an increased risk of developing cancer [27, 29–32]. While there have been no reports that PDGF and BMP-7 demonstrate this exact side effect profile, PDGF’s clinical use has been limited by its short half-life and oncogenicity, while BMP-7’s use has been limited by its cost [33, 34]. Thus, research investigating alternative treatment options for improved fracture healing and vascularization is ongoing. One avenue of novel therapies that has recently been explored is stimulating endogenous megakaryocytes (MKs) with the main MK growth factor, thrombopoietin (TPO) [35], and other cytokines that MKs produce.

Previous studies have shown that mice that displayed an increased number of BM-residing MKs also displayed an increase in cortical bone thickness and trabecular bone number, and had an increased risk of developing osteosclerosis [36–39]. MKs have been shown to exert these effects on bone structure by mechanisms that involve both direct cell-to-cell contact and paracrine secretion of cytokines [25, 40]. MK’s paracrine effects may influence bone growth and homeostasis through a variety of molecular mechanisms such as increasing osteoblast proliferation, suppressing osteoclastogenesis, and increasing the vessel-forming activity of endothelial cells (ECs) [25, 41]. Their effect on EC activity has thus far been attributed to the production and release of VEGF and TGF-β1 [25, 42]. If the production of these cytokines and other angiogenic growth factors were to be altered in MKs due to aging, this could provide a possible mechanism that contributes to the delayed vascularization of fracture calluses that is seen in elderly populations. MKs from older mice have previously been shown to have a decreased capacity to support osteoblast proliferation [43], but the effects of aging on the ability of MKs to stimulate angiogenesis have not yet been characterized.

To investigate the effects of aging on the angiogenic capacity of MKs through paracrine signaling, we measured the difference in activity of murine BM endothelial cells (BMECs) that were supplemented with media cultured with MKs from young (3–4-month-old) and aged (22–24-month-old) mice for 3 days. The activity of BMECs was measured by their capacity to proliferate, develop vessel-like structures, migrate, and express angiogenic factors.

## RESULTS

### Proliferation of BMECs when cultured with MK CM

**Figure 1:**
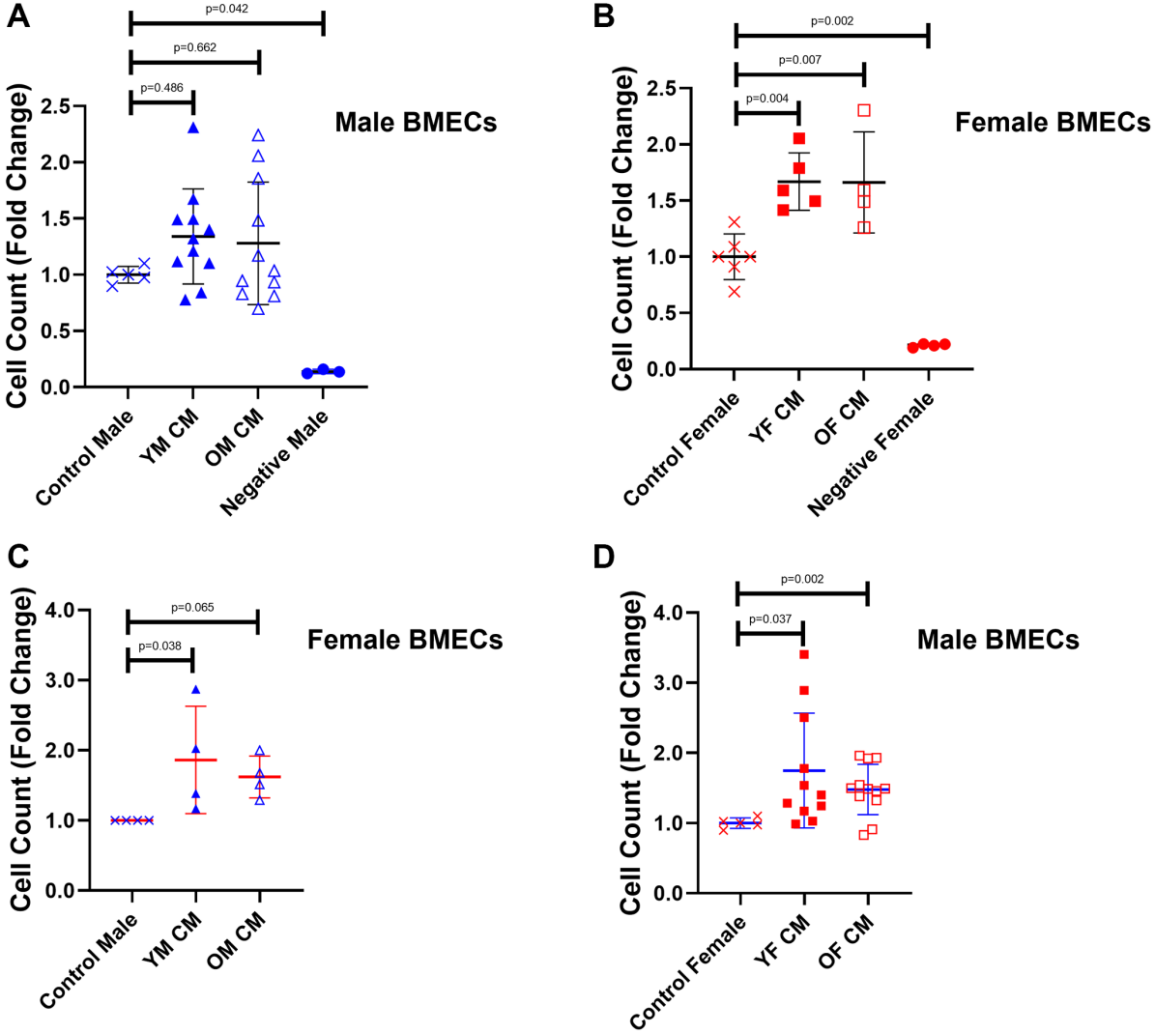
**Proliferation of male and female BMECs increases with female MK CM treatment.** (**A**) Male BMECs treated with male MK CM. (**B**) Female BMECs treated with female MK CM. (**C**) Female BMECs treated with male MK CM. (**D**) Male BMECs treated with female MK CM. The number of cells was quantified following a 48-hour incubation period. Data are expressed as a mean ± SD fold change relative to their respective controls (*n* = 3–11 biological replicates/group). For the negative control group (α-MEM lacking serum), *n* = 3 for male and 4 for female BMECs). Significance was determined using a one-way ANOVA with Tukey’s post-hoc test (**A**, **B**, **D**) or Kruskal-Wallis test (**C**) depending on the normalcy of the data distribution as determined by a Shapiro-Wilk test. Female BMECs treated with young female MK CM, old female MK CM, and young male MK CM proliferated significantly more than control female BMECs. Male BMECs treated with young or old female MK CM exhibited significantly greater proliferation. No significant differences in proliferation were found in male BMECs treated with male MK CM.

First, we examined cell growth as a proxy for the angiogenic potential of MK conditioned media (CM) on BMECs (Figure 1). While supplementation of young and aged male MK CM did not significantly impact the male BMEC cell number (Figure 1A), female BMEC proliferation was significantly improved with female MK CM, irrespective of age, compared to control female BMECs (Figure 1B). When treated with MK CM generated from mice of the opposite sex, male BMECs exhibited a significant increase in proliferation when cultured with young and old female MK CM (Figure 1D); female BMECs, however, only showed significantly greater proliferation when cultured with young male MK CM (Figure 1C). Together, these results suggest that female MKs secrete factors that increase both male and female BMEC proliferation. Notably, male MK CM generated from young mice significantly increased female BMEC proliferation and male MK CM generated from old mice showed a trending increase in female BMEC, whereas no significant or trending differences were observed when young or aged male MK CM was cultured with male BMECs. It should be noted that throughout this manuscript we report significant and trending differences due to the variability inherent in the use of primary cell cultures.

### Effects of MK CM on vessel-like structure formation of BMECs

**Figure 2:**
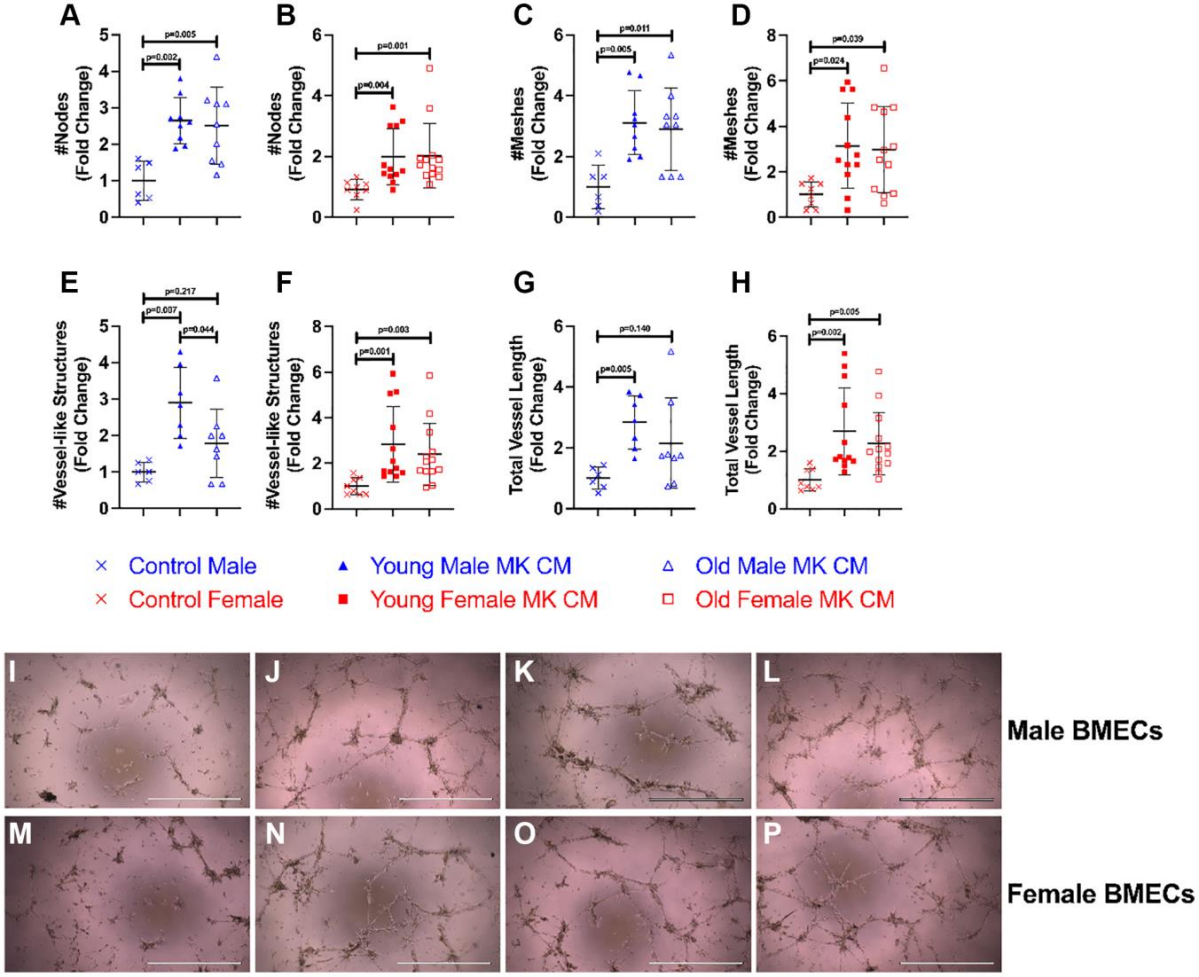
**Various MK CM treatments increase length and complexity of BMEC vascular network.** The number of nodes (**A**, **B**), the number of meshes (**C**, **D**), the number of vessel-like structures formed (**E**, **F**), and the total length of vessel-like structures (**G**, **H**) were quantified following an 8-hour incubation period. Representative images of BMEC vessel-like formation assays for control media (**I**, **M**), young MK CM (**J**, **N**), old MK CM (**K**, **O**), and positive control (**L**, **P**), with males in panels I-L, and females in panels (**M**–**P**). Data are expressed as a mean ± SD fold change relative to their respective controls (*n* = 8–13 biological replicates/group). Significance was determined using one-way ANOVA with Tukey’s post-hoc analysis (**A**, **C**, **D**, **E**) or Kruskal-Wallis test with Dunn’s post-hoc analysis (**B**, **F**, **G**, **H**) depending on the normalcy of the data distribution as determined by a Shapiro-Wilk test. Both young and aged female MK CM significantly increased all vessel-like formation properties. While young male MK CM also improved vessel-like properties, aged male MK CM did not increase the number of vessel-like structures nor the length of the vessel-like structures.

The effects of young and aged MK CM on the angiogenic potential of BMECs were evaluated by vessel-like structure formation (Figure 2). The criteria for what constitute a vessel-like structure are found in the Methods section. Vessel-like structure formation in the control and treatment groups was assessed and compared by the number of nodes, number of meshes, number of vessel-like structures, and total vessel length. As shown in Figure 2A–2H, treatment with young MK CM significantly improved all parameters in both males and females. Treatment with aged MK CM, irrespective of sex, significantly improved node and mesh numbers (Figure 2A–2D), and aged female MK CM significantly improved the number and length of vessel-like structures (Figure 2F, 2H, 2M–2O). On the other hand, aged male MK CM resulted in a trending, but not statistically significant, increase in the number of vessel-like structures (*p* = 0.22) and total vessel length (*p* = 0.14) (Figure 2E, 2G, 2I–2K). When comparing young and aged sex-matched MK CM, a significant difference between young and aged male MK CM on the number of vessel-like structures was detected (*p* = 0.044), where young male MK CM-treated groups had significantly more vessel-like structures than groups treated with aged male MK CM (Figure 2E). Images of positive control media (5% FBS) cultured with male and female BMECs are found in Figure 2L, 2P, respectively.

**Figure 3:**
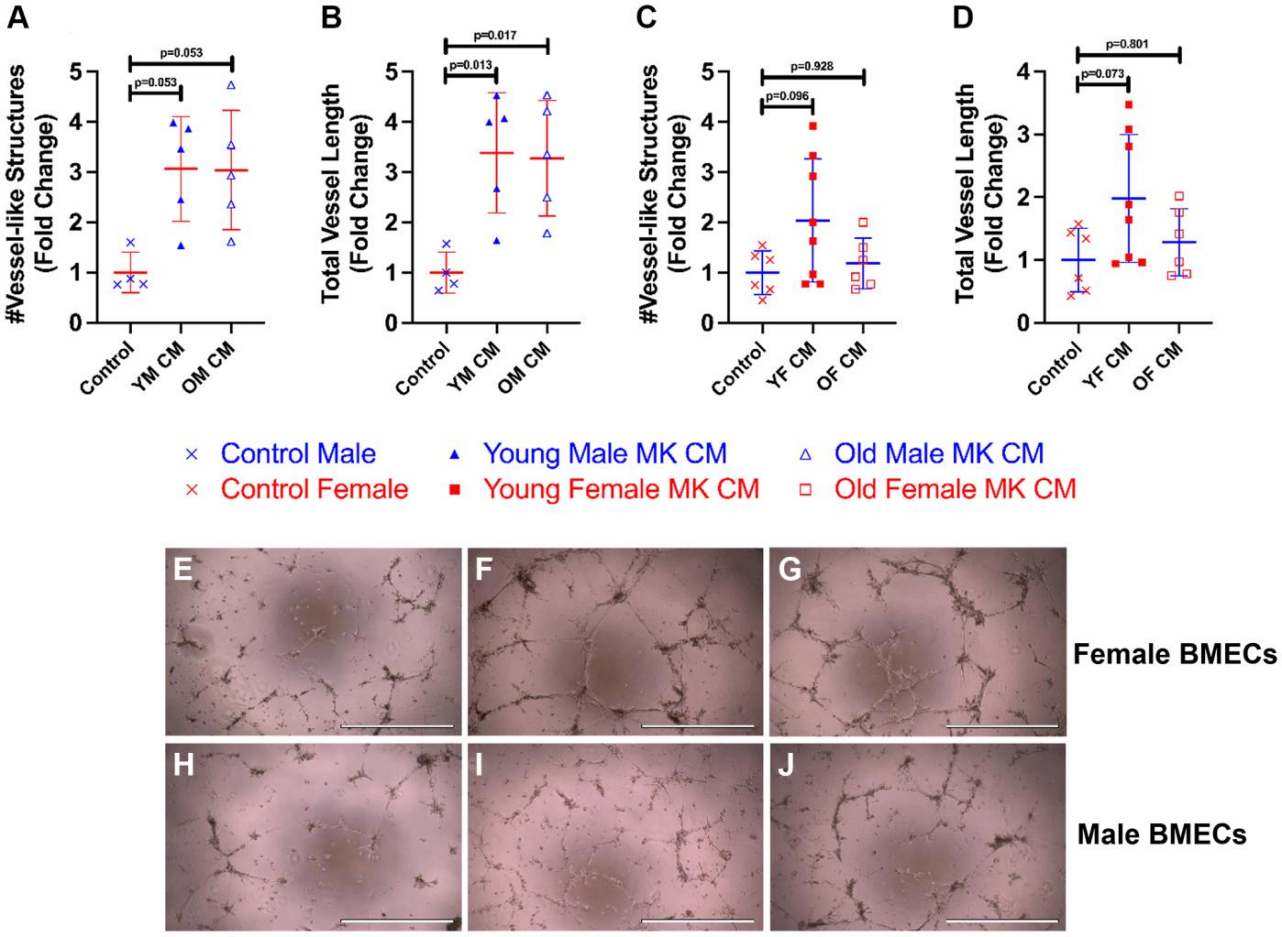
**Young and old male MK CM significantly increase female BMEC vessel-like structure length.** The number of vessel-like structures formed and total length of vessel-like structures were quantified following an 8-hour incubation period for female BMECs treated with male MK CM (**A**, **B**) and male BMECs treated with female MK CM (**C**, **D**). Representative images of BMEC vessel-like formation assays for Control media (**E**, **H**), young MK CM (**F**, **I**), and old MK CM (**G**, **J**), treatment groups are also shown for both female BMECs treated with male MK CM (**E**–**G**) and male BMECs treated with female MK CM (**H**–**J**). Data are expressed as a mean ± SD fold change relative to their respective controls (*n* = 4–8 biological replicates/group). Significance was determined using one-way ANOVA with Tukey’s post-hoc analysis (**B**–**D**) or Kruskal-Wallis test with Dunn’s post-hoc analysis (**A**) depending on the normalcy of the data distribution as determined by a Shapiro-Wilk test.

To examine how treatment of MK CM generated from mice of the opposite sex affects male and female BMEC tube formation, we also treated male BMECs with female MK CM from young or old mice and female BMECs with male MK CM from young or old mice (Figure 3). Interestingly, young and old male MK CM increased total vessel length of female BMECs and demonstrated a strong trend toward increasing total number of vessels (Figure 3A, 3B, 3E–3G). However, male BMECs did not respond to old female MK CM but did show a trend toward increased vessel number and length when treated with young female MK CM (Figure 3C, 3D, 3H–3J). Overall, the data in Figures 2 and 3 primarily support the idea that treatment of BMECs with MK CM, especially young MK CM, improves their capacity for vessel-like structure formation irrespective of the sex of the mice from which MKs were generated.

### Effects of MK CM on BMEC unidimensional chemotaxis

**Figure 4:**
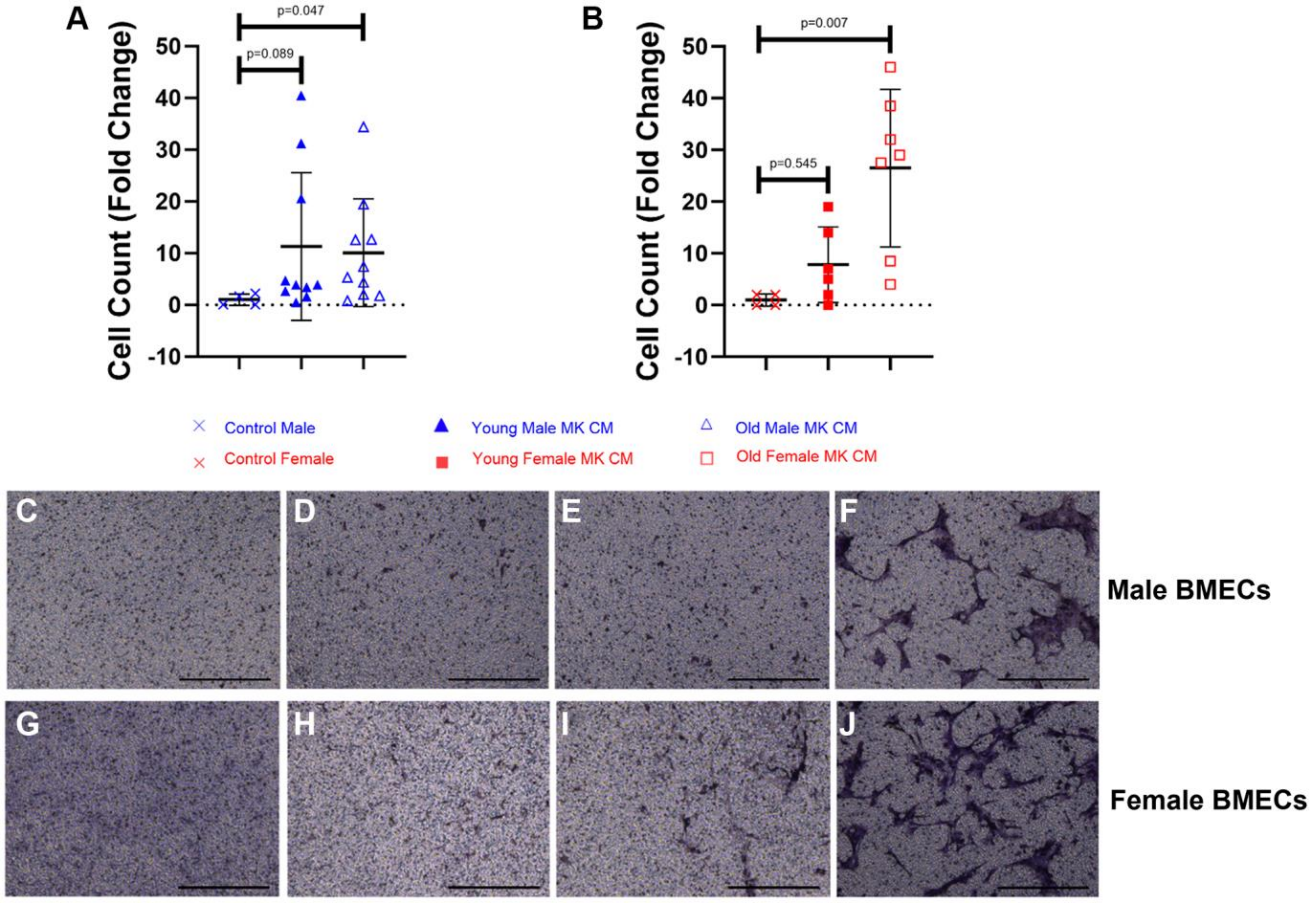
**Aged female and male MK CM increase unidimensional, transwell migration of BMECs.** The number of migrated male BMECs (**A**) and female BMECs (**B**) was quantified following an overnight incubation period. Data are expressed as a mean ± SD fold change relative to their respective controls (*n* = 4–10 biological replicates/group). Significance was determined using a one-way ANOVA with Tukey’s post-hoc test. Aged MK CM significantly increased the migration of BMECs compared to control regardless of sex. Representative images of male BMECs using crystal violet staining (**C**- Control, **D**- Young Male MK CM, **E**- Old Male MK CM, **F**- 5% FBS control). Representative images of female BMECs using crystal violet staining (**G**- Control, **H**- Young Female MK CM, **I**- Old Female MK CM, **J**- 5% FBS control).

The impacts of MK CM treatment on BMEC unidimensional chemotaxis were analyzed using transwell migration assays. As shown in Figure 4, the young male MK CM treatment elicited a trending increase in male BMEC migration, and the aged male MK CM significantly increased male BMEC migration (*p* = 0.047) compared to control male media (Figure 4A, 4C–4E). Similarly, female BMECs that were treated with aged female MK CM showed significantly more migration through the transwell membrane compared to control BMECs (*p* = 0.007, Figure 4B, 4G–4I). Images of positive control media (5% FBS) for male and female BMECs are found in Figure 4F, 4J, respectively. In general, aged MK CM promoted unidimensional migration of BMECs as compared to that observed with young MK CM.

### Effects of MK CM on BMEC two-dimensional motility

**Figure 5:**
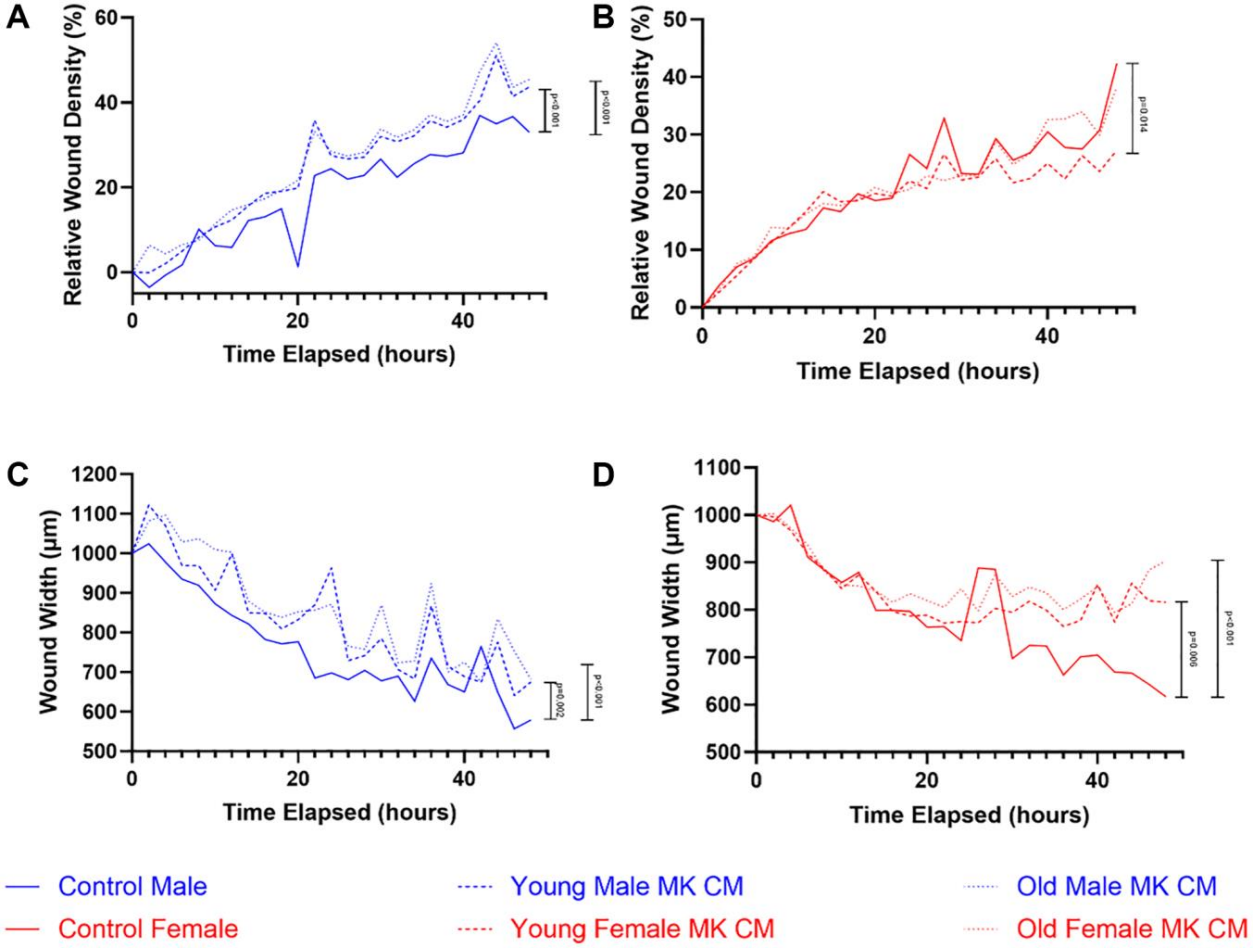
**Altered two-dimensional motility of BMECs with MK CM treatments.** The changes in relative wound density (**A**-males, **B**-females) and wound width (**C**-males, **D**-females) were quantified through data collected every 2 hours over a 48-hour period. Significance was determined using a two-way ANOVA with Dunnett’s post-hoc test (significance bars are noted to the right on all panels). Cross-sectional analyses were made at 12-, 24-, and 48-hour timepoints for all panels as well. No significant differences in relative wound density (**A**, **B**) were observed in any cross-sectional analyses. A one-way ANOVA found that female BMEC wound closure (panel **D**) was significantly inhibited when treated with female aged MK CM at the 48-hour timepoint (*p* = 0.029), but all other cross-sectional analyses were not significant in panels **C** and **D**. Solid, dashed, and dotted lines represent the means (*n* = 3–16 biological replicates/group).

The effects of young and aged MK CM treatment on BMEC two-dimensional motility were examined through a wound migration assay, which compared the treatment and control groups in terms of the relative wound density of cells within the produced wound and the wound width over 48 hours (Figure 5). While relative wound density increased over time for all 3 groups, male MK CM treatment in male BMECs and control treatment in female BMECs led to greater relative wound density when compared by 2-way ANOVA; no significant differences were observed between groups specifically at 12, 24, or 48 hours (Figure 5A, 5B). When wound width was examined, control media resulted in the greatest decrease in both male and female BMECs compared to the MK CM groups, compared by 2-way ANOVA (Figure 5C, 5D). Similarly, no significant differences were observed between the groups specifically at 12, 24, or 48 hours for wound width in males. However, there was a significant difference between aged female and control wound width (*p* = 0.029) at 48 hours.

### MK CM alters gene expression of angiogenic and inflammatory factors in BMECs

**Figure 6:**
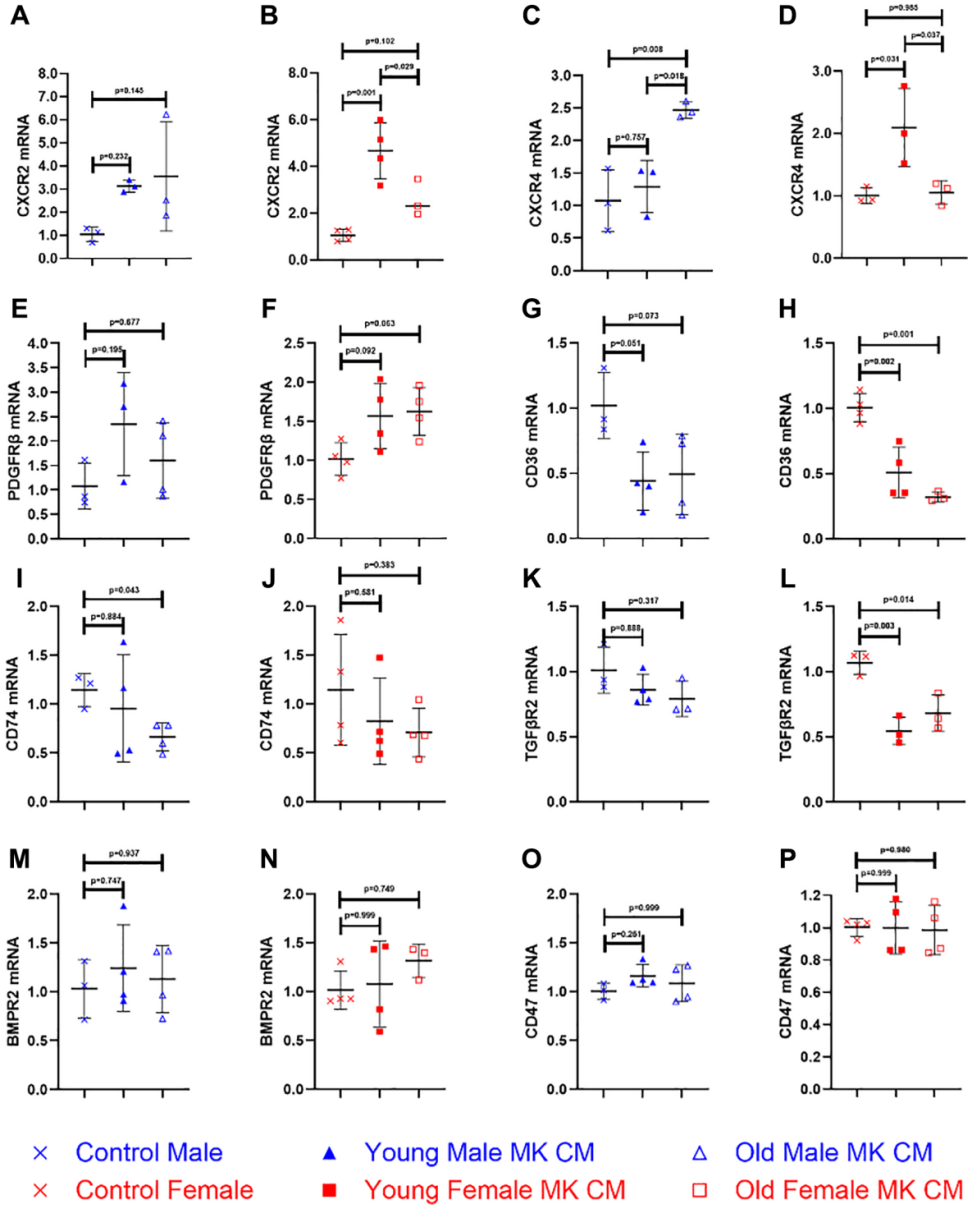
**Certain MK CM treatments alter various genes of BMECs to a proangiogenic and proinflammatory phenotype.** The changes in mRNA expression were quantified following an overnight incubation period. Data are expressed as a mean ± SD fold change relative to their respective controls with post-hoc *p*-values listed in each panel (*n* = 3–4 biological replicates/group). GAPDH was used as an internal control. Significance was determined using one-way ANOVA with Tukey’s post-hoc analysis or Kruskal-Wallis test with Dunn’s post-hoc analysis depending on the normalcy of the data distribution as determined by a Shapiro-Wilk test. MK CM treatments showed trending or significant increases in BMEC expression of CXCR2 (**A**, **B**) and PDGFRβ (**E**, **F**), while showing trending or significant decreases of CD36 (**G**, **H**), CD74 (**I**, **J**), and TGFβR2 (**K**, **L**) expression. Although aged male and young female MK CM significantly increased CXCR4 expression, young male and aged female MK CM did not (**C**, **D**). No significant differences in gene expression levels were seen in BMPR2 (**M**, **N**) or CD47 (**O**, **P**).

Further understanding of how MK CM affects BMECs was pursued. While the previous experiments analyzed BMEC functionality on a cellular level, we sought to examine the molecular changes as well. Eleven genes of receptors present in BMECs were examined for their relative mRNA expression (Figure 6 and Supplementary Figure 1). The selection process was based on the known presence of specific factors in MK CM. These factors may bind to the gene’s encoded receptors and have a downstream effect of changing the degree of transcription, thereby influencing the proangiogenic and antiangiogenic properties of the BMECs [44, 45].

Of the eleven genes examined, five were found to have at least one type of MK CM exhibit significantly altered expression relative to controls. Both young and old male MK CM increased BMEC expression of CXCR2 (Figure 6A) though not significantly. Young female MK CM significantly increased CXCR2 expression, relative to both control and aged female MK CM (Figure 6B). Aged male MK CM significantly increased expression of CXCR4 (Figure 6C) not only relative to control, but also compared to young male MK CM. The opposite was true for CXCR4 with female MK CM, as young female MK CM significantly increased its expression relative to both control and aged female MK CM treatment (Figure 6D). Aged female MK CM increased expression of PDGFRβ to a nonsignificant degree, which was found to have mirroring trends in young female and young male MK CM treatments for this gene (Figure 6E, 6F). Young and aged female MK CM significantly decreased expression of CD36, while male MK CM showed trending decreases (Figure 6G, 6H). Aged male MK CM reduced expression of CD74 to a significant extent, while young male, young female, and old female MK CM treatments showed no significant change in expression of CD74 (Figure 6I, 6J). Female MK CM, both young and aged, decreased expression of TGFβR2 significantly while male MK CM treatments decreased its expression to a nonsignificant degree (Figure 6K, 6L). BMPR2 and CD47 expression remained unchanged (Figure 6M–6P), as did all other genes in Supplementary Figure 1.

## DISCUSSION

The risk of bone fractures increases with aging due to a variety of factors including decreased production of androgens and estrogens, decreased dietary intake of calcium and vitamin D, decreased osteoblast activity, and an increasingly sedentary lifestyle [4, 46, 47]. Moreover, bone diseases that are observed in aging populations such as osteoporosis and osteomalacia can lead to increased risk of fracture. MKs may play a role in these consequences of aging as they have been shown to increase in frequency and decrease in functional ability to stimulate bone production with age [43]. MKs and the factors they secrete have been established in recent decades to be prime regulators of the bone marrow microenvironment. While we have previously described in detail their ability to influence osteoblasts, osteoclasts, the hematopoietic niche, and several aging-associated bone disorders, this research is the first attempt at identifying changes that occur in the MK secretome due to aging and sex that contribute to effects on bone vasculature. Future studies are warranted to understand specific factors, signaling pathways, and mechanisms that explain our reported effects [48].

Notably, the relationship between MKs, angiogenesis, and ECs had not been fully described. Although numerous MK-secreted factors are known to influence EC functionality, few studies have directly examined the impact of MKs on the regulation of ECs. Tang et al. [25] conducted a vessel-like formation assay which found increased vessel lengths when ECs were treated with MK CM. Use of a TGF-β inhibitor decreased MK CM-induced vessel lengthening. It was also determined that increasing HIF-1α expression in ECs is one mechanism by which MK CM induces EC expression of VEGF. The effects of aging on MK CM and other aspects of EC functionality have not been examined to the best of our knowledge.

Our study examined BMECs isolated from young male and female mice when treated with young or aged MK CM *in vitro*. We analyzed the proliferation, formation of vessel-like structures, unidimensional and two-dimensional motility of these cells in response to the different MK CM, and changes observed in mRNA expression of receptors in BMECs in response to the different MK CM. This study aimed to understand the general effects of MK CM on BMECs, identify the differences between young and aged MK CM, and determine if these differences may potentially account for the changes in BMEC function with aging. A secondary goal of this study was to determine if any observable differences between sexes could be discerned. Sex-based differences in bone homeostasis and fracture healing have been well-established. Although the primary discussion regarding these differences typically focuses on the roles of estrogen and other sex hormones, sex-based differences in osteoblasts and BMEC responses to VEGF that result in divergent bone physiology have also been described [49].

The ability of MKs to secrete factors that upregulate BMEC proliferation and angiogenesis has been implicated through its known secretion of VEGF [42]. Moreover, patients with prolonged isolated thrombocytopenia have significantly reduced levels of VEGF secretion coupled with reduced BMEC counts, further suggesting that MK CM affects BMEC growth [50]. Here, we further support the assertions that MKs secrete factors that increase the proliferation of BMECs. Specifically, the BMECs from both sexes treated with young or old female MK CM exhibited significant increases in cell number compared to when they received control treatment (Figure 1B, 1D). When male BMECs were treated with male MK CM, only non-significant increases were observed (Figure 1A); female BMECs, however, proliferated significantly more with young male MK CM treatment and almost reached significance with old male MK CM treatment (Figure 1C). This may suggest that in terms of proliferation, female BMECs are more responsive to MK-secreted factors irrespective of the sex from which MKs were isolated. Furthermore, male MKs may also secrete different factors, or the factors may be generated at different concentrations than those generated by female MKs. Finally, it is also formally possible that the expression of receptors binding MK CM may be altered. Discriminating between these possibilities requires further experimentation.

We also analyzed four parameters in the vessel-like formation assay, also referred to as tube formation assay: nodes, meshes, number of vessel-like structures, and total vessel length. When examining our results, it becomes evident that MK CM stimulates BMEC vessel-like formation to an extent, as expected [25]. Female BMECs treated with young or aged MK CM of both sexes improved all assayed parameters of vessel-like formation (Figures 2 and 3). Young female MK CM caused trending increases in male BMEC vessel length and number (Figure 3C, 3D). Male BMECs treated with aged male MK CM saw only increases in the number of nodes and meshes and had fewer vessel-like structures than the young male MK CM counterparts (Figure 2A, 2C, 2E, 2G). Nodes and meshes are measurements of vessel-like structure interconnectedness, indicating that both young and aged male MK CM may maintain the vasculature complexity of BMECs. However, a significant decrease in the number of vessel-like structures between the young and aged MK CM-treated male groups demonstrates that the capability of MKs to induce BMECs vascular formation may be decreased with aging (Figure 2E). Reduced angiogenesis and vascular density have been characterized as consequences of aging [51]. While further examination is warranted, it is possible that this may be attributed partially to MK CM aging changes, specifically in males.

EC migration is a critical component of angiogenesis, particularly during the fracture healing process [52, 53]. When aged MK CM was used as a chemoattractant, significant increases in BMEC unidimensional or transwell migration were observed, while the young MK CM treatments showed trending increases. On the other hand, the two-dimensional, wound migration assay, identified divergent results. The calculation of relative wound density is the degree of cellular density found in the wound relative to the degree of cellular density found in the undamaged region surrounding the wound. Thus, it accounts for cells found in the wound that may have been present not only due to migration, but due to proliferation as well. There was a significant increase in relative wound density compared to control when male BMECs were treated with young or old male MK CM. Perplexingly, control media in the female BMECs resulted in greater relative wound density than young female MK CM. Wound width was significantly larger in BMECs treated with MK CM regardless of sex or age compared to control groups. This may initially seem to directly contradict the results of the transwell assay; however, the mechanisms of the two assays are important to note. Transwell assay examines BMECs transversing across a membrane toward a chemoattractant found in the underlying media. It is not directly supplemented by MK CM. On the other hand, wound migration assays directly supplement the BMECs with MK CM. The differences between the results of these two assays may be attributed to the variation in migratory behavior of BMECs in unidimensional and two-dimensional environments. Given the designs of each study type, the distribution of the chemotactic agent is different, and this could be a reason for some of the observed disparity in the two results. Overall, the migratory effects of MK CM on BMECs are complex and may hold the key to primary mechanisms by which EC functionality changes with aging [54].

Examining gene expression changes may further elucidate underlying mechanisms. Here we examined eleven genes. Expression patterns for three of the eleven genes analyzed are found in Supplementary Figure 1. FLT-1 and KDR are VEGF receptors 1 and 2, respectively. FLT-1 has shown contrasting results regarding its effect on ECs; while it has been described as antiangiogenic, particularly in embryonic contexts, it has also shown proangiogenic properties [55, 56]. Primarily, soluble FLT-1 functions as a regulator of EC sprouting. Hypoxia reduces soluble FLT-1, which inhibits VEGF, thus creating a directional growth to the hypoxic tissue due to uninhibited VEGF [57]. KDR has also shown different results, although it has not been as clearly characterized as being antiangiogenic [58, 59]. ITGβ1, the integrin β1 receptor, has been shown to inhibit EC proliferation but promote sprouting and migration [60]. While these factors are significant components of EC function, their expression with MK CM treatment remained unchanged. Two other genes of interest, BMPR2 and CD47, found in Figure 6 also lack significant differences across all groups. While BMPs and their receptors are typically thought of as osteoblast and osteoclast regulators, they are also found on ECs and can influence their growth [61]. However, MK CM did not affect the expression of BMPR2, despite the known secretion of BMPs by MK CM [48]. CD47, also known as integrin-associated protein and a possible regulator of EC senescence, showed no differences [62, 63].

CXCR2 and CXCR4 are CXC chemokine receptors and typically regarded to be inducers of angiogenesis [64]. CXCR2 is a receptor of growth-related oncogenes (GRO)-α, β, and γ, as well as neutrophil-activating peptide (NAP)-2, epithelial neutrophil-activating peptide (ENA)-78, and interleukin (IL)-8. These factors have all been found to be promoters of EC growth, migration, and angiogenesis [64–66]. We found a significant increase in CXCR2 expression in young female MK CM treatment, compared to both control and aged female MK CM. CXCR4 is the receptor for stromal-cell derived factor (SDF)-1, known for its proangiogenic properties specifically in BMECs [67]. CXCR4 expression was among the more surprising results: while aged male MK CM significantly increased its expression, young male MK CM showed no change and was in fact significantly reduced when compared to aged male MK CM. The opposite was found in the females, with aged female MK CM showing no differences and young female MK CM significantly increasing BMEC expression of CXCR4. At this time, differences between males and females in their MK secreted factor components has not been fully described. What is known is MKs express estrogen receptors, and estrogen has been shown to promote MK maturation and potentially increase their proliferation [48, 68]. Moreover, estrogen increases MK expression of osteoprotegerin (OPG) and reduces RANKL expression [69]. Despite this, the differences in MK CM effects with regards to CXCR4 and its ligand SDF-1 should be elucidated further.

Some of the genes assessed presented clearer results. PDGFRβ is the receptor for PDGF-BB and is an established proangiogenic receptor [70]. Both young and aged female MK CM and young male MK CM caused trending elevation in its expression. It is interesting to note the correlation between the expression of PDGFRβ and the results of the unidimensional, transwell assay: PDGF-BB is a chemoattractant that induces EC motility, so it may be the primary factor involved in transwell migration of the BMECs [71]. CD36 is a thrombospondin-1 receptor and is typically regarded to be an inhibitor of angiogenesis [72]. Its expression was significantly decreased in both female MK CM treatments and exhibited trending decreases in both male MK CM treatments. The increases in vessel-like formation may be particularly attributed to reductions in CD36 expression, as there were significant improvements in all parameters with exception of aged male MK CM treatment.

CD74 was observed to have trending reductions in its expression with both female MK CM and a significant decrease upon old male MK CM treatments. CD74 is the receptor for the proinflammatory mediator, macrophage migration inhibitory factor (MIF). Although MIF is proangiogenic and most other proinflammatory receptors have shown increases, it is possible that MK secretion of MIF is upregulated to such an extent that it reduces the expression of CD74 through a negative feedback mechanism [45, 73]. TGFβR2 showed similar results, with trending reductions in male BMECs and significant reductions in female BMECs. Just as with CD74, TGFβR2 is a proangiogenic receptor, and its reduced expression typically results in impaired vasculogenesis [74]. Thus, it may be that MKs secrete high levels of TGF-β, and its abundant presence in MK CM may lead to negative feedback inhibition. TGFβR2, in particular, has been shown to be downregulated with increased TGF-β expression [44].

The interpretation of RNA expression, as it currently stands, requires further substantiation. All the genes analyzed herein were those of BMEC receptors, and receptor expression may increase or decrease for different reasons that are often contrasting. CD36 transcription regulation, by example, is contingent on a diverse array of extracellular signaling such as IL-4 and TGF-β, in addition to non-coding RNAs and intracellular mechanisms [75]. The abundance of a factor and its ability to influence the cell’s functionality may result in upregulation or downregulation of its receptor through various feedback mechanisms. This is a logical future direction. Furthermore, comparing the bone vascular niches as well as our gene panel in young and aged male and female mice *in vivo* may support our findings and elucidate mechanisms behind our observed differences *in vitro*.

In conclusion, we have reported various similarities and differences in the effects of MK CM on BMEC biology based on age and sex, which aligns with the growing literature that MK secreted factors alter the bone marrow microenvironment (Table 1). Specifically, we found that treatment with all tested categories of MK CM (young, aged, male, female) increased the tendency of BMECs to form vessel-like structures, undergo unidimensional migration, upregulated the expression of the proangiogenic gene CXCR2, and downregulated the expression of CD36. We also found that female MK CM increased the proliferation of BMECs while male MK CM showed only an effect on female BMECs, and MK CM generally decreased the two-dimensional motility of BMECs. It is important to note that this reduction in wound migration found with MK CM treatment, as well as some of the aging and sex-based differences in gene expression, particularly found in CXCR4, should be further examined. An understanding of which factors regulate which mechanisms of EC functionality may allow for isolation of one or a few factors that influence EC migration changes with aging, resulting in the development of targeted therapy to improve EC migration, subsequent angiogenesis, and fracture healing.

**Table 1:** Results summary.

	**Young male MK** **CM**	**Old male MK** **CM**	**Young female MK** **CM**	**Old female MK** **CM**
Proliferation	−	−	↑↑	↑↑
Nodes	↑↑	↑↑	↑↑	↑↑
Meshes	↑↑	↑↑	↑↑	↑↑
# Vessel-like Structures	↑↑	↑	↑↑	↑↑
Total Vessel Length	↑↑	↑	↑↑	↑↑
1-Dimensional Migration	↑	↑↑	−	↑↑
Relative Wound Density	↑↑	↑↑	↓↓	−
Wound Width	↑↑	↑↑	↑↑	↑↑
CXCR2 Expression	↑	↑	↑↑	↑
CXCR4 Expression	−	↑↑	↑↑	-
PDGFRβ Expression	↑	−	↑	↑
CD36 Expression	↓	↓	↓↓	↓↓
CD74 Expression	−	↓↓	−	−
TGFβR2 Expression	−	↓	↓↓	↓↓
BMPR2 Expression	−	−	−	−
CD47 Expression	−	−	−	−
ITGβ1 Expression	−	−	−	−
FLT-1 Expression	−	−	−	−
KDR Expression	−	−	−	−

↑↑/↓↓ = statistically significant. ↑/↓ = trending, not statistically significant.

## MATERIALS AND METHODS

### Animals

Young (3–4-month-old) and old (22–24-month-old), male and female C57BL/6J mice were purchased from Jackson Laboratory or were generously provided by the National Institute of Aging (NIA), respectively. The Indiana University School of Medicine Institutional Animal Care and Use Committee approved all described studies.

### EC isolation from BM

BMECs were isolated from the BM of femurs, tibiae, and humeri of all mice. Briefly, after euthanasia, skin, muscle, and soft tissue were stripped from each of the bones, after which the distal and proximal epiphyses of the bones were removed. All bones were individually placed into sterile, punctured 0.5 mL tubes that were inserted into 1.5 mL tubes. Each 1.5 mL tube contained 750 μL α-MEM (Gibco, Grand Island, NY, USA) with 10% fetal bovine serum (FBS, Biowest, Riverside, MO, USA). BM was isolated from the bones by centrifugation at 14,000 g for 2 minutes at 4°C. The extracted BM pellets were then resuspended in 1 mL of Complete EC Growth Media (ScienCell, Carlsbad, CA, USA), which contained EC growth supplements, 7.5% FBS, and 1% penicillin/streptomycin (Gibco, Grand Island, NY, USA). The resuspended pellets were plated in a 12-well plate coated with 4 μg/mL fibronectin (Thermo Fisher Scientific, Waltham, MA, USA) containing an additional 1 mL of Complete EC Growth Media. Media changes were performed every two days and BMECs were cultured for 5–7 days prior to use in experimental assays.

### MK CM preparation

Murine BM from C57BL/6J mice was extracted and plated in Dulbecco’s Modified Eagle Medium (DMEM; Gibco, Grand Island, NY, USA), supplemented with 5% FBS (Hyclone, Logan, UT, USA), 1% penicillin/streptomycin/glutamine (Gibco, Grand Island, NY, USA), and conditioned medium from a TPO producing cell line [38] until numerous large MKs were observed. MKs were then isolated from the other cell population using a one-step Bovine Serum Albumin (BSA, Millipore-Sigma, Burlington, MA, USA) gradient [38]. The isolated MKs were removed from the bottom of the gradient, washed with Phosphate Buffered Saline (PBS, Gibco, Grand Island, NY, USA), and re-suspended in phenol-free α-MEM at 1 million cells/ml for 3 days. The CM was collected, centrifuged, and stored at −80°C until use.

### Proliferation assay

BMECs were seeded in a 96-well plate (Corning, Corning, NY, USA) at 1 × 10^3^ cells/well with 25% MK CM isolated from young or old mice of both sexes (all containing 1.25% FBS), or 25% α-MEM (Control Media) containing 1.25% FBS, or α-MEM with 0% FBS (Negative control) and were incubated for 1 to 2 days to assess proliferation. The BMECs were fixed with 5% neutral buffered formalin (NBF) at room temperature for 15 minutes. The fixed BMECs were stained with 0.05% crystal violet for 20 minutes and were subsequently washed with tap water prior to an overnight drying period. The EVOS FL Cell Imaging System was utilized to image BMEC cultures, and ImageJ.1.52a software was used for counting analysis.

### Vessel-like structure formation assay

**Figure 7:**
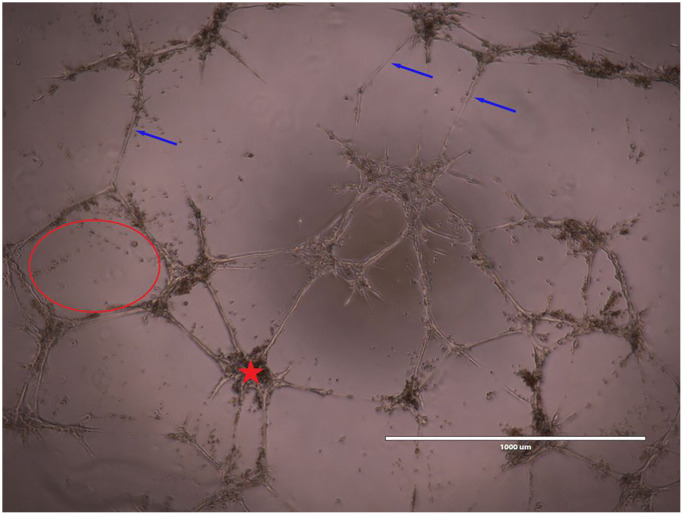
**Representative image of BMEC vessel-like formation assay.** Images were taken at 10x magnification. Meshes (red outline) and nodes (red star) were automatically analyzed. Meshes were defined as areas enclosed by three or more nodes and nodes were defined as a point where two or more vessels intersect. A blinded analyzer manually measured the number of vessel-like structures (indicated by blue arrows) as well as vessel length.

96-well plates (Corning Inc., Corning, NY, USA) were chilled in a −20°C freezer overnight. They were subsequently coated with 50 μL/well of Matrigel basement membrane matrix (Corning, Corning, NY, USA) and incubated 45 minutes at 37°C prior to plating and culturing BMECs. BMECs were seeded at 1 × 104 cells/well with their respective treatments and incubated for 8 hours prior to cell imaging (EVOS FL Cell Imaging System). α-MEM (Control group), 25% MK CM isolated from young or old mice of both sexes (all containing 1.25% FBS), and α-MEM with 5% FBS (positive control) were the designated groups. Vessel-like structure formation was quantified with ImageJ.1.52a software. Images are taken at 10× magnification. The number of nodes, number of meshes, number of complete vessels, and total vessel-like structure length were the parameters analyzed and an example is shown in Figure 7 [30]. Briefly, meshes were defined as areas enclosed by three or more nodes, and nodes were defined as a point where two or more vessels intersect. Simple Neurite Tracer, a plugin of ImageJ.1.52b software, was employed for the manual measurement of the total vessel length and number of vessel-like structures. A blinded analyzer manually measured the number of vessel-like structures as well as vessel length. The automated Angiogenesis Analyzer, a plugin of ImageJ.1.52a software, was used to analyze the number of meshes and nodes.

### Transwell assay or unidimensional chemotaxis assay

500 μL of 25% MK CM or 25% α-MEM was added to the bottom of each well in 24-well plates; α-MEM with 5% FBS was used as a positive control. A transwell membrane (8 μm pore size, Corning, Corning, NY, USA) was placed over the media. Cells resuspended in Basal EC Growth Media (ScienCell, Carlsbad, CA, USA containing only 7.5% FBS and 1% penicillin/streptomycin solution. Basal EC Growth Media lacked EC growth supplements.) were seeded on the top of the membrane at a density of 4 × 10^5^ cells/well. The plates were placed in an incubator at 37°C for a period of 24 hours. The membranes were fixed with 5% NBF at room temperature for 15 minutes. The membranes were stained with 0.5% crystal violet for 20 minutes and dried using sterile cotton tip applicators (Puritan Medical Products, Gulliford, ME, USA). The EVOS FL Cell Imaging System was used to image the migration of the BMECs across the membrane at a magnification of 10×. Analysis of images was completed with the ImageJ.1.52a software.

### Wound migration assay

96-well plates (Corning, Corning, NY, USA) were seeded with BMECs placed in Complete EC Growth Media at a density of 1 × 10^5^ cells/well and incubated at 37°C for a period of 24 hours to achieve 100% confluence. Complete EC Growth Media was aspirated and replaced with 25% MK CM or 25% α-MEM. The IncuCyte^®^ WoundMaker (Essen BioScience, Ann Arbor, MI, USA) generated a wound in the center of every well. The IncuCyte ZOOM^®^ Live-Cell Analysis System (Essen BioScience, Ann Arbor, MI, USA) took images of each well every 2 hours at 10X magnification for 48 hours. Relative wound density and wound widths were compared across the entire timecourse and specifically at 12, 24, and 48 hours. The IncuCyte^™^ Scratch Wound Cell Migration Software (Essen BioScience, Ann Arbor, MI, USA) analyzed the images taken.

### RNA isolation and gene expression

BMECs were seeded in a 6-well plate at a concentration of 1 × 10^6^ cells/well with 25% MK CM or 25% α-MEM. The plates were incubated at 37°C for 24 hours to achieve no less than 80% confluency. The RNeasy Mini kit (QIAGEN, Hilden, Germany) was used for total RNA isolation. The Transcriptor First Strand cDNA Synthesis Kit (Roche, Basel, Switzerland) was used to prepare cDNA, which was then analyzed through quantitative PCR using the Power SYBR^™^ Green PCR Master Mix (Thermo Fisher Scientific, Waltham, MA, USA) on a CFX96 Touch Real-Time PCR Detection System (Bio-Rad, Hercules, CA, USA). GAPDH was used as an internal control. The fold change in gene expression was obtained through the 2^−ΔΔCT^ method. Several genes were analyzed. Table 2 lists the genes and primers used.

**Table 2:** Primer pairs for gene expression.

**Gene**	**Forward sequence**	**Reverse sequence**
CXCR2	CTCTATTCTGCCAGATGCTGTCC	ACAAGGCTCAGCAGAGTCACCA
CXCR4	GACTGGCATAGTCGGCAATGGA	CAAAGAGGAGGTCAGCCACTGA
PDGFRβ	CATCCGCTCCTTTGATGATCTT	GTGCTCGGGTCATGTTCAAGT
CD36	GGACATTGAGATTCTTTTCCTCTG	GCAAAGGCATTGGCTGGAAGAAC
CD74	CGCGACCTCATCTCTAACCAT	ACAGGTTTGGCAGATTTCGG
TGFβR2	CCTACTCTGTCTGTGGATGACC	GACATCCGTCTGCTTGAACGAC
BMPR2	AGAGACCCAAGTTCCCAGAAGC	TCTCCTCAGCACACTGTGCAGT
CD47	TGCGGTTCAGCTCAACTACTG	GCTTTGCGCCTCCACATTAC
ITGβ1	CTCCAGAAGGTGGCTTTGATGC	GTGAAACCCAGCATCCGTGGAA
FLT1	CCACCTCTCTATCCGCTGG	ACCAATGTGCTAACCGTCTTATT
KDR/VEGFR2	TTTGGCAAATACAACCCTTCAGA	GCTCCAGTATCATTTCCAACCA
GAPDH	CGTGGGGCTGCCCAGAACAT	TCTCCAGGCGGCACGTCAGA

### Statistics

Statistical analyses and data normality calculations were performed with GraphPad Prism (ver. 8 and 10, GraphPad Software, La Jolla, CA, USA). Results are displayed in figures as mean ± SD or line graphs. Data in this manuscript comparing 3 or more groups were analyzed by one-way analysis of variance (ANOVA) with Tukey-Kramer post hoc test for parametric data. A Kruskal-Wallis test with a Dunn’s multiple comparison post-hoc test was used for nonparametric data. For the wound healing assay, a two-way ANOVA with a Dunnett’s Multiple comparison test was used to examine the main treatment effects. Specific post-hoc *p*-values are displayed on the graphs, and a *p*-value of less than 0.05 was considered significant.

## Supplementary Materials

Supplementary Figure 1
