# Supplementary material for: The effects of young and aged, male and female megakaryocyte conditioned media on angiogenic properties of endothelial cells
Source: Aging (Albany NY). 2024 Nov 22;16(21):13181–200. doi: 10.18632/aging.206077 (PMC11719103; doi:10.18632/aging.206077)
Supplement: Supplementary Figure 1 [file aging-16-206077-s001.pdf]

SUPPLEMENTARY FIGURE

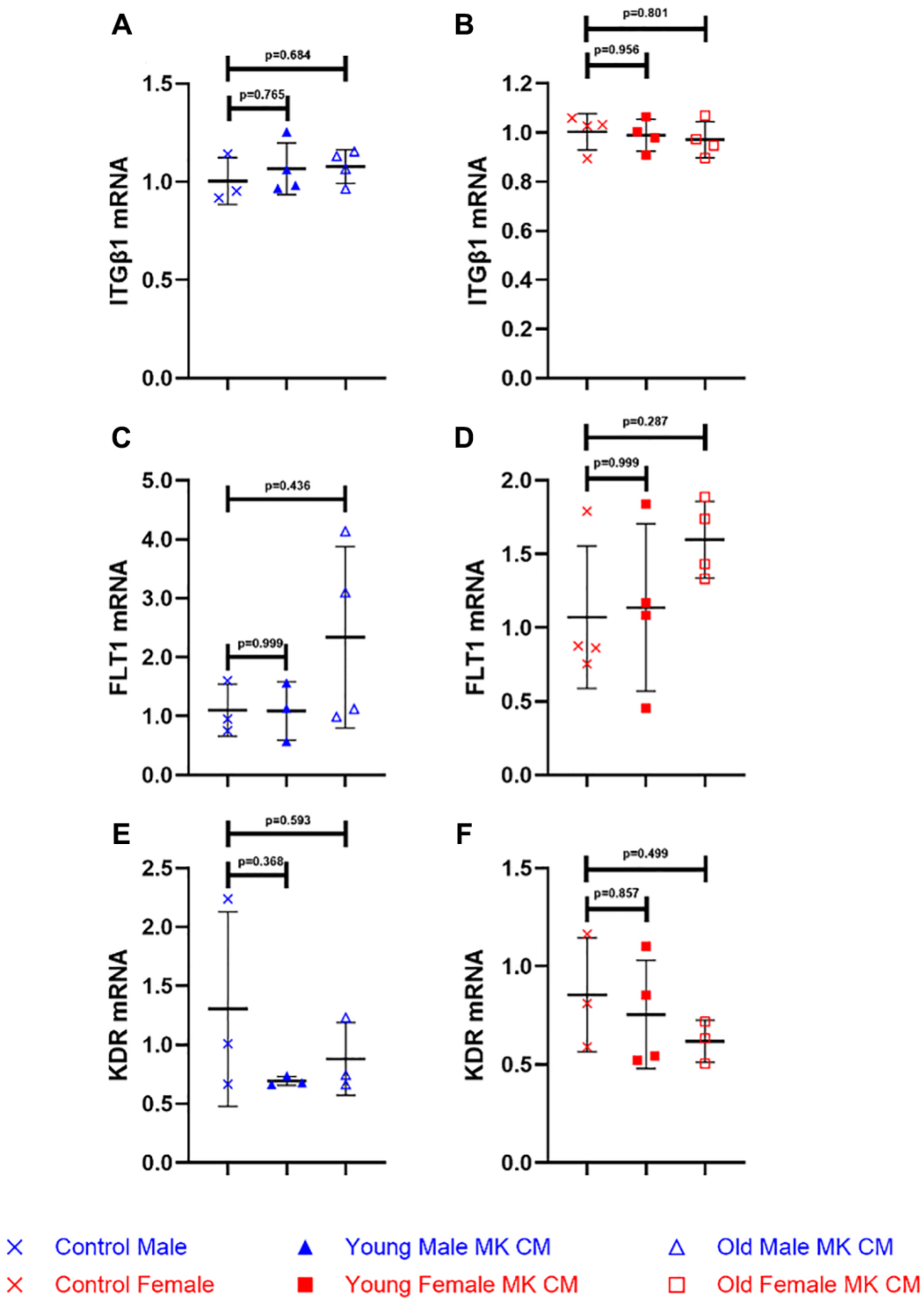

Supplementary Figure 1. MK CM did not alter BMEC expression of several genes. ITGβ1 (**A**, **B**), FLT-1 (**C**, **D**), and KDR (**E**, **F**) expression were not changed in BMECs by MK CM treatments. The changes in mRNA expression were quantified following an overnight incubation period. Data are expressed as a mean ± SD fold change relative to their respective controls with post-hoc *p*-values listed in each panel (*n* = 3–4 biological replicates/group). GAPDH was used as an internal control. Significance was determined using one-way ANOVA with Tukey's post-hoc analysis or Kruskal-Wallis test with Dunn's post-hoc analysis depending on the normalcy of the data distribution as determined by a Shapiro-Wilk test.
